# Adults with cerebral palsy exhibit uncharacteristic cortical oscillations during an adaptive sensorimotor control task

**DOI:** 10.1038/s41598-024-61375-x

**Published:** 2024-05-11

**Authors:** Erica H. Hinton, Morgan T. Busboom, Christine M. Embury, Rachel K. Spooner, Tony W. Wilson, Max J. Kurz

**Affiliations:** 1 Institute for Human Neuroscience, Boys Town National Research Hospital, Omaha, NE USA; 2 Center for Pediatric Brain Health, Boys Town National Research Hospital, Boys Town, NE USA; 3 Department of Pharmacology and Neuroscience, Creighton University, Omaha, NE USA; 4 Institute for Human Neuroscience, Boys Town National Research Hospital, 14090 Mother Teresa Lane, Boys Town, NE 68010 USA

**Keywords:** Neuroimaging, Brain imaging, Magnetoencephalography, Dexterity, Hand, Motor cortex, Neonatal brain damage

## Abstract

Prior research has shown that the sensorimotor cortical oscillations are uncharacteristic in persons with cerebral palsy (CP); however, it is unknown if these altered cortical oscillations have an impact on adaptive sensorimotor control. This investigation evaluated the cortical dynamics when the motor action needs to be changed “on-the-fly”. Adults with CP and neurotypical controls completed a sensorimotor task that required either proactive or reactive control while undergoing magnetoencephalography (MEG). When compared with the controls, the adults with CP had a weaker beta (18–24 Hz) event-related desynchronization (ERD), post-movement beta rebound (PMBR, 16–20 Hz) and theta (4–6 Hz) event-related synchronization (ERS) in the sensorimotor cortices. In agreement with normative work, the controls exhibited differences in the strength of the sensorimotor gamma (66–84 Hz) ERS during proactive compared to reactive trials, but similar condition-wise changes were not seen in adults with CP. Lastly, the adults with CP who had a stronger theta ERS tended to have better hand dexterity, as indicated by the Box and Blocks Test and Purdue Pegboard Test. These results may suggest that alterations in the theta and gamma cortical oscillations play a role in the altered hand dexterity and uncharacteristic adaptive sensorimotor control noted in adults with CP.

## Introduction

Cerebral palsy (CP) is an umbrella term for a group of posture and movement disorders that result from an initial disturbance to the developing brain^1^. Although the initial disturbance is seen as being non-progressive, there are often cascading neurophysiological changes that affect the overall fidelity of the upper extremity motor actions. The majority of the research evaluating these neurophysiological changes have centered on youth with CP, with far less attention paid towards the adult population^2^. This leaves a substantial knowledge gap in our understanding of the life course of individuals with CP, which is problematic as most individuals with CP have a life expectancy well beyond 58 years^3^. Hence, there is a need for foundational knowledge on the long-term effects of the initial perinatal brain injuries on the adult sensorimotor system. Such insights have the potential to redirect or alter the course of the current neurologically-based treatment approaches that are being used to improve upper extremity motor function in adults with CP.

It is well established that the production of motor actions involve time–frequency dependent changes in the oscillatory activity of the sensorimotor cortical neurons^4,5^. Specifically, the sensorimotor cortices exhibit a robust beta (15–30 Hz) event-related desynchronization (ERD) several hundred milliseconds prior to onset of the motor action that is sustained throughout the course of the motor action^6–14^. This beta ERD is accompanied by a transient theta (4–7 Hz) and gamma (70–90 Hz) event-related synchronization (ERS) that is tightly yoked with the onset of the motor action^15–18^. Upon the completion of the motor action, there is a resynchronization of beta oscillations or a post-movement beta rebound (PMBR)^7,10,11,19,20^. The consensus is that the beta ERD is associated with planning of the motor action, while the gamma and theta ERS are associated with the execution and timing of the motor command. The PMBR is presumed to be associated with motor inhibition and/or assessment of the sensory feedback that is returned to the cortex after the motor action is complete^21–23^.

Several investigations have shown that the beta ERD, gamma ERS and PMBR tend to be weaker when individuals with CP plan and execute their hand motor actions^24–26^. Furthermore, the extent of the deviations seen in the beta and gamma sensorimotor cortical oscillations appear to be connected with slower reaction times, deviations in muscular performance, and the extent of motor execution errors in those with CP^12,24–28^. The altered cortical oscillations have been suggested to reflect inaccuracies in the neural calculations of the feedforward motor command. However, there are cases where the motor command needs to be adjusted to account for last second changes in the timing of the motor action to meet the task demands. It is currently unknown if the seminal deviations in the sensorimotor cortical oscillations have subsequent effects on the ability of persons with CP to adjust the motor command in real-time.

When it comes to adaptive motor control, most studies have focused on proactive or reactive inhibitory control^29^. Proactive control is when adjustments in the motor command are foreseen ahead of time, while reactive control is when the motor command must be adjusted in the moment^30^. The stop-no go task is often used to tease out the effects of proactive versus reactive^31–35^. However, these studies have focused on the proactive and reactive mechanisms that contribute to motor inhibition. What has been studied in less detail is the simple on-the-fly adjustment of movement parameters. Having to adjust the timing of the onset of a motor action, as opposed to suppressing the initiation of a motor action, is much more common in real-life motor behavior. For example, when picking up an object, real-time adjustments need to be made depending on size, weight, etc. Numerous behavioral studies have shown that persons with CP have difficulty anticipating the necessary grip forces to pick up objects^36^, and require a longer time to plan sequential movements during an object manipulation task^37^. Furthermore, persons with CP tend to employ a stereotypical grip pattern when reaching for an object and lack the ability to alter their grip in order to adapt to the changing task demands^38–43^. Altogether these behavioral results imply that persons with CP lack the ability to reactively alter their initially planned motor actions.

There is mounting evidence that the sensorimotor cortical oscillations are uncharacteristic in persons with CP^13,24,25,27,28,44^. However, whether these cortical abnormalities have an impact on their reactive motor control has yet to be established. Therefore, the objective of this investigation was to evaluate the sensorimotor cortical oscillations of adults with CP when the timing of the motor action needs to be changed “on-the-fly.” To that end, we used MEG to image the cortical dynamics as neurotypical (NT) controls and adults with CP performed a hand motor task that required either proactive or reactive sensorimotor control. Based on the prior literature in youth with CP, we hypothesized that the sensorimotor cortical dynamics associated with the production of a hand motor action would be weaker in adults with CP when compared with NT controls. We also hypothesized that the sensorimotor cortical oscillations would be further deviant for the reactive condition when compared with the proactive condition. Lastly, we hypothesized that the extent of the cortical aberrations would be connected with the reaction time and the clinical assessments of the hand’s dexterity. Testing of these hypotheses will provide new insights on the nature of the uncharacteristic motor actions seen in adults with CP. Subsequently, these new insights will provide a new platform for the development of neuroscience informed treatment strategies that are lacking in the physical and occupational literature for persons with CP^45–47^.

## Methods

### Ethical approval

The study protocol conformed with the standards set by the *Declaration of Helsinki*. The protocol was approved by the Institutional Review Board at Boys Town National Research Hospital. Informed consent was acquired from all the participants.

### Participants

Nineteen adults with CP who had a spastic presentation (Age = 32.05 ± 11.24 years; Gross Motor Function Classification Score (GMFCS) I–IV)^48^; Manual Ability Classification System (MACS) I–IV)^49^ and nineteen neurotypical (NT) controls (Age = 30.21 ± 9.93 years) with no neurological or musculoskeletal impairments participated in this investigation. Further details on the participants with CP is provided in Table S1 of the Supplement. The GMFCS and MACS were used for the enrollment criteria because they are the most widely utilized clinical assessments for quantifying the extent of the impairments seen in persons with CP for databases, clinical research, and program evaluation^49,50^. A participant with a GMFCS level II has difficulty walking on uneven surfaces and has decreased walking speed. While a participant with a GMFCS IV has severely limited mobility and primarily relies on a wheelchair for walking long distances. The MACS levels are similar in that a person with a level II handles objects with reduced quality and speed, while a person at level IV handles objects with limited success. There were no differences in age, sex, race or handedness between groups (Ps > 0.05). The participants with CP had not undergone upper extremity surgeries, had no botulinum toxin injections in the past year, and were not on anti-spastic medications.

### MEG acquisition and experimental paradigm

**Figure 1:**
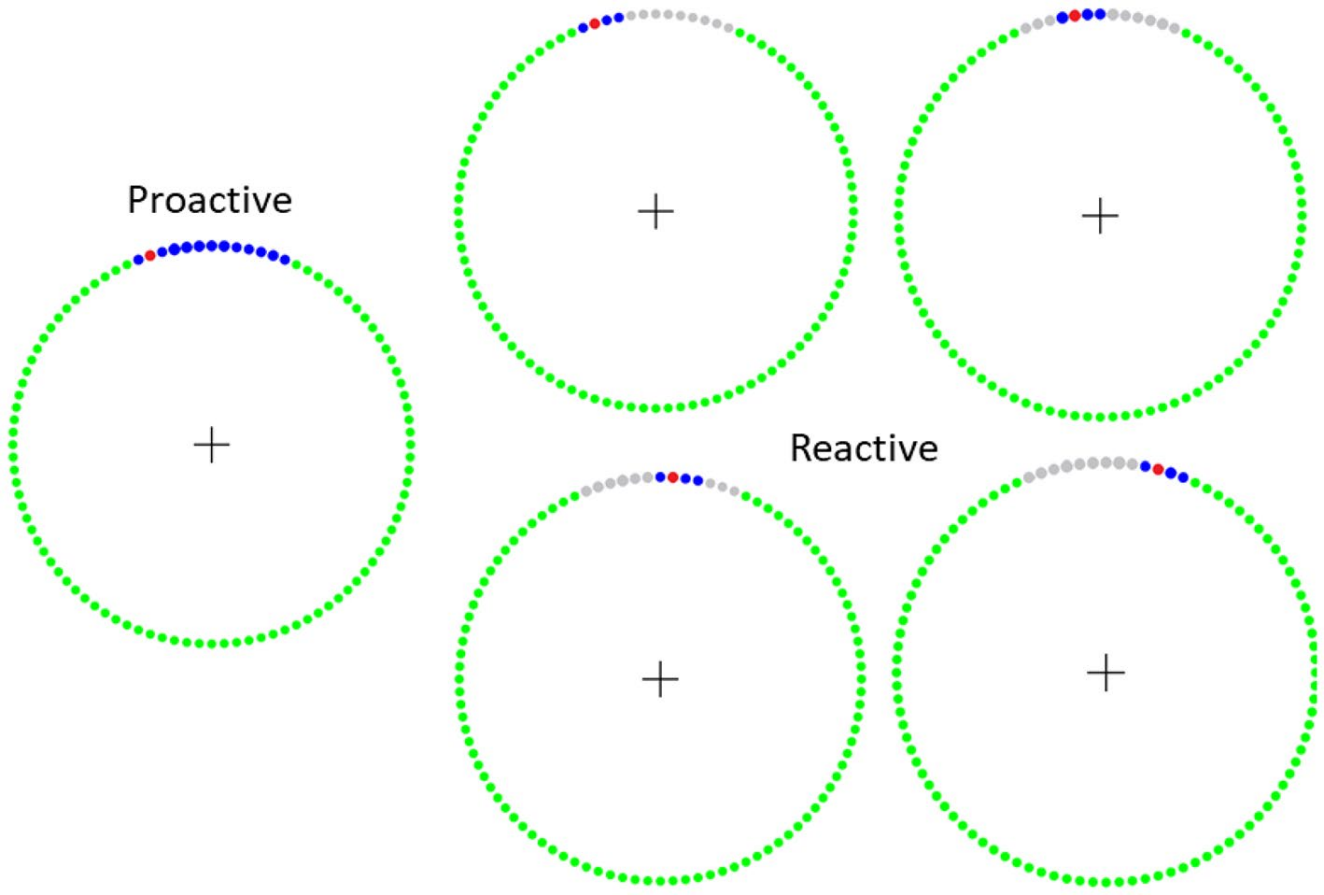
Experimental Paradigm. Graphical representation of the proactive and reactive conditions where the participant performed a button press as soon as the red dot entered the blue target interval of the circle. The green circle is always shown on the screen as the participant views the red dot traversing the circle in a clockwise fashion. The proactive condition is shown on the left and the reactive condition had four possible circumstances (middle and right) where 150 ms before the red dot entered the blue interval the target would shrink and shift within the original interval to one of four fixed locations shown in the respective panels. Further details on the experimental paradigm are provided in the text.

All recordings were conducted using a whole head MEG system (MEGIN/Elekta, Helsinki, Finland) that was in a one-layer magnetically shielded room with active shielding engaged for advanced environmental noise compensation. The neuromagnetic responses during the experiment were sampled continuously at 1 kHz with an acquisition bandwidth of 0.1–330 Hz. The experiment consisted of a clock-based proactive–reactive finger-tapping task to analyze dynamic neural mechanisms serving the adaptive control of voluntary movement, while keeping movement kinematics, motor selection and planning processes constant^30^. For this task, individuals responded the same for both the proactive and reactive conditions, which allowed for more straightforward interpretations of adaptive cueing mechanisms. The participant maintained fixation on a centrally located crosshair while a red dot traversed a circle in a clockwise direction every five seconds (Fig. 1) under two conditions: (1) Proactive and (2) Reactive. The diameter of the circle was 0.21 m and was displayed on a back projection screen that was ~ 1 m from the participant. The red dot traversed the circle at a rate of 0.13 m/s. Both conditions had a blue target interval where the participant was instructed to perform a button press with the right index finger as quickly and accurately as possible as soon as the red dot entered the target interval. In the proactive condition, the blue target interval was fixed near the 12 o’clock position (Fig. 1). For the reactive conditions, at approximately 150 ms before the red dot entered the blue interval the target would shrink and shift within the original interval to one of four fixed locations (Fig. 1). Every participant completed 100 proactive and 100 reactive trials, and target interval locations were presented in a pseudorandom order. Furthermore, the presentation of either the proactive or reactive conditions were randomized. The total time to complete the experiment was ~ 15 min. Prior to the start of the experiment the participants practiced exemplary proactive and reactive conditions to ensure that they understood the task requirements.

### MEG pre-processing and source imaging

Prior to the experiment, four coils were affixed to the participant’s head and the location of the coils, three fiducial points, and the scalp surface were digitized (Fastrak, Polhemus Navigator Sciences, Colchester, VT, USA). During the MEG recording, an electric current with a unique frequency label (e.g., 322 Hz) was fed to each of the coils and was used to localize the head in reference to the MEG sensors. The participant’s MEG data were subsequently co-registered with the MRI and transformed into standardized space.

Each participant’s MEG data were individually corrected for head motion that occurred during the task performance using the MaxFilter software (MEGIN/Elekta). In addition, the signal space separation method with a temporal extension was used for noise reduction^51^. All the MEG data pre-processing, co-registration and source imaging was performed with Brain Electrical Source Analysis (BESA) software (BESA v7.1; Grafelfing, Germany). Artifact rejection was based on an individualized fixed threshold method supplemented with visual inspection. The continuous magnetic time series were divided into epochs of 4500 ms duration, with 0 ms defined as movement onset and the baseline being − 2000 to − 1500 ms window. Epochs containing artifacts were rejected based on an individualized fixed threshold method, supplemented with visual inspection. The artifact-free epochs for each sensor were transformed into the time–frequency domain using complex demodulation and averaged over the respective trials. These sensor-level data were normalized to the mean power during the baseline, and the specific time–frequency windows selected for source imaging were determined by statistical analysis of the sensor-level spectrograms across the entire array of gradiometers from all participants^52–54^. Based on these time–frequency windows, a minimum variance vector beamformer based on the cross-spectral densities was used to calculate the source power across the entire brain volume per participant at a 4.0 mm^3^ resolution^55^, and the source power in these images were normalized per subject using a separately averaged pre-stimulus noise period of equal duration and bandwidth^56,57^.

The peak voxels identified in the grand-averaged beamformer images were used for extracting virtual sensor neural time courses by applying the sensor weighting matrix derived through the forward computation to the preprocessed signal vector^7,54^. The neural time courses were subsequently transformed into the time–frequency domain and the source orientation with the strongest response was selected for further analyses. The average across the window of interest was the primary outcome measure and this was used to assess for condition and group differences.

### Upper extremity motor behavioral performance assessments

Along with the MEG data, the output of the button pad was simultaneously collected at 1 kHz. The reaction time was calculated as the difference between the press onset relative to when the red dot entered the target window. The mean of the motor performance and the coefficient of variation of the motor performance were the primary outcome variables.

The participants also completed the Box and Blocks and Purdue Pegboard assessments of hand dexterity. The Purdue Pegboard Test is a standardized test that assesses the total number of pegs picked up and placed into the holes of a board within a 30-s period. The Box and Blocks Test is also a standardized test that involves the participant moving as many blocks as possible from one compartment across to another within a 60-s period using the tested limb^58–60^.

Lastly, the participants completed the wrist position test, which quantifies the ability of the participant to correctly identify the joint’s position following a movement performed by the examiner^61,62^. The test consists of 20 predetermined wrist flexion and extension angles, where the examiner passively moved the wrist to the endpoint angular position as the participant’s vision of the wrist position was occluded. The participants subsequently indicated the perceived wrist angle by aligning an arrow pointer towards the imposed wrist joint position. The perceived angle indicated by subjects was compared to the imposed angle to determine position sense error (in degrees). The mean absolute error over the 20 positions was used as an index of proprioceptive discrimination ability^61^.

### Statistical analysis

To evaluate differences in the strength of cortical oscillations and motor behavior, we performed separate 2 × 2 mixed model ANOVAs with condition (proactive or reactive) as a within subjects’ factor and group (NT and CP) as a between subjects’ factor. Pearson correlations were also used to determine the relationship between the motor performance behavioral variables and the strength of cortical oscillatory responses. Correlations were done separately for each group (CP and NT), as well as condition (proactive or reactive). All statistical analyses were performed in JASP with a 0.05 alpha level.

## Results

### Motor behavioral results

**Figure 2:**
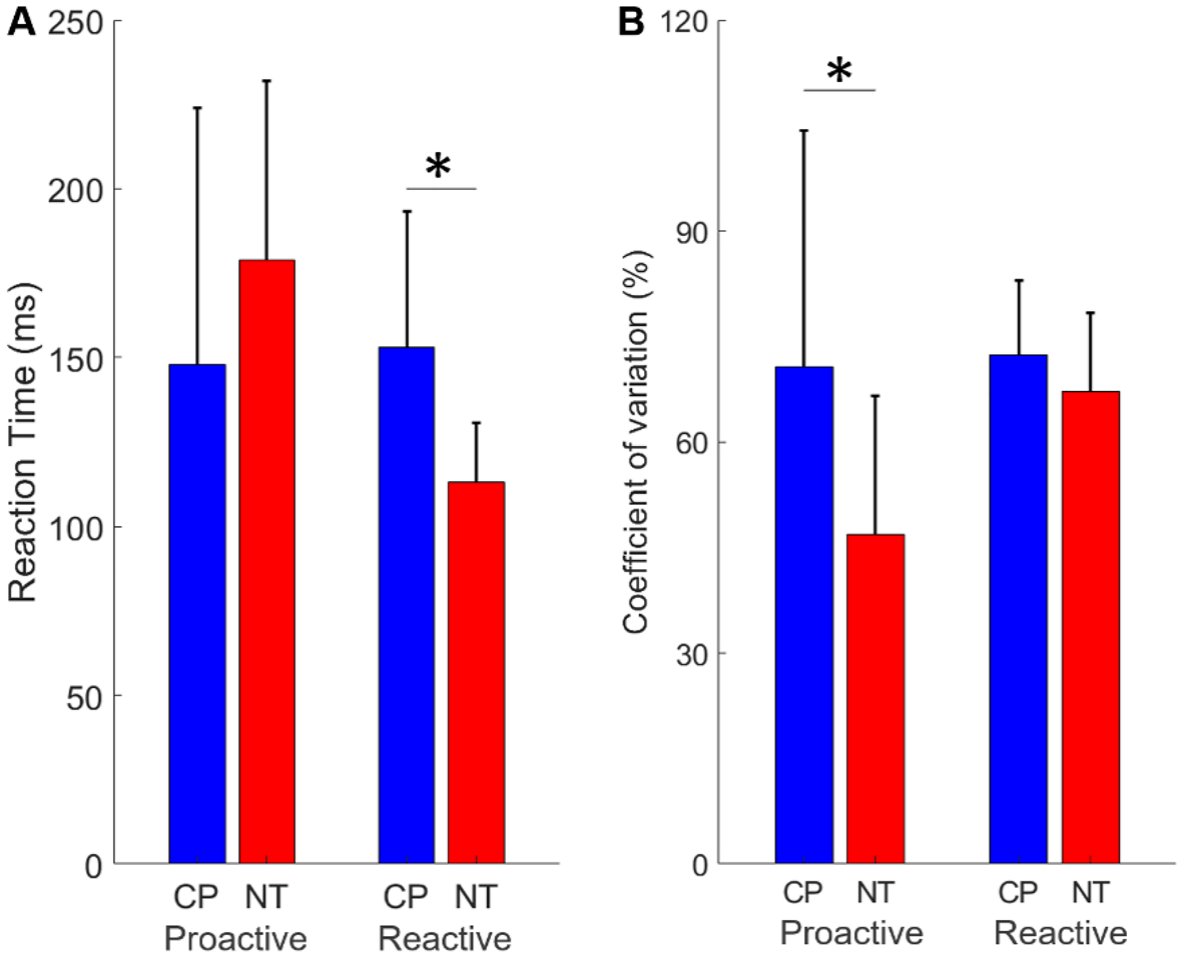
Motor performance during the task. (**A**) As shown, the adults with cerebral palsy (CP) responded slower during the reactive, but not the proactive condition compared to the neurotypical controls (NT). (**B**) The onset of the button press was more variable in the adults with CP in the proactive condition when compared to NT. The bar graph results are shown as the mean ± the standard deviation. *p < 0.05.

In regard to behavioral performance during the proactive–reactive sensorimotor task (i.e., button press in ms from target onset), we observed a significant main effect of condition (Proactive = 163.21 (66.63) ms, Reactive = 132.99 (36.68) ms, F = 6.674, p = 0.014), but not a main effect of group (CP = 150.43 (60.12) ms, NT = 145.87 (51.30) ms, F = 0.149, p = 0.702). There was also a significant condition by group interaction for reaction time (F = 9.207, p = 0.004). The post-hoc analyses indicated that the adults with CP were less precise during the reactive trials (CP = 153.08 (40.08) ms, NT = 112.91 (17.55) ms, F = 16.015, p = 0.0003), but that their reaction time was similar to controls during the proactive trials (CP = 147.79 (76.22) ms, NT = 178.84 (52.99) ms, F = 0.154, p = 0.154; see Fig. 2A).

For the coefficient of variation, there was a significant main effect of condition (Proactive = 58.76 (29.70) %, Reactive = 69.81 (10.98) %, F = 8.292, p = 0.007) and there also was a main effect of group (CP = 71.51 (24.61) %, NT = 57.06 (18.76) %, F = 6.665, p = 0.014). Lastly, there was a significant interaction between condition and group (F = 5.783, p = 0.021). Post-hoc analysis indicated that the adults with CP had greater variability in their motor performance for the proactive trials (C = 70.60 (33.67) %, NT = 46.92 (19.57) %, F = 7.024, p = 0.012), but not for the reactive trials (CP = 72.42 (10.48) %, NT = 67.20 (11.11) %, F = 2.220, p = 0.150; see Fig. 2B).

### Cortical oscillations at the sensor and source level

There was not a significant effect of group (CP = 177.84 ± 11.87, NT = 181.84 ± 11.93) (F = 0.919, p = 0.344) as well as condition (Proactive = 90.79 ± 6.37, Reactive = 89.24 ± 6.37) (F = 3.42, p = 0.073) on number of MEG trials accepted.

**Figure 3:**
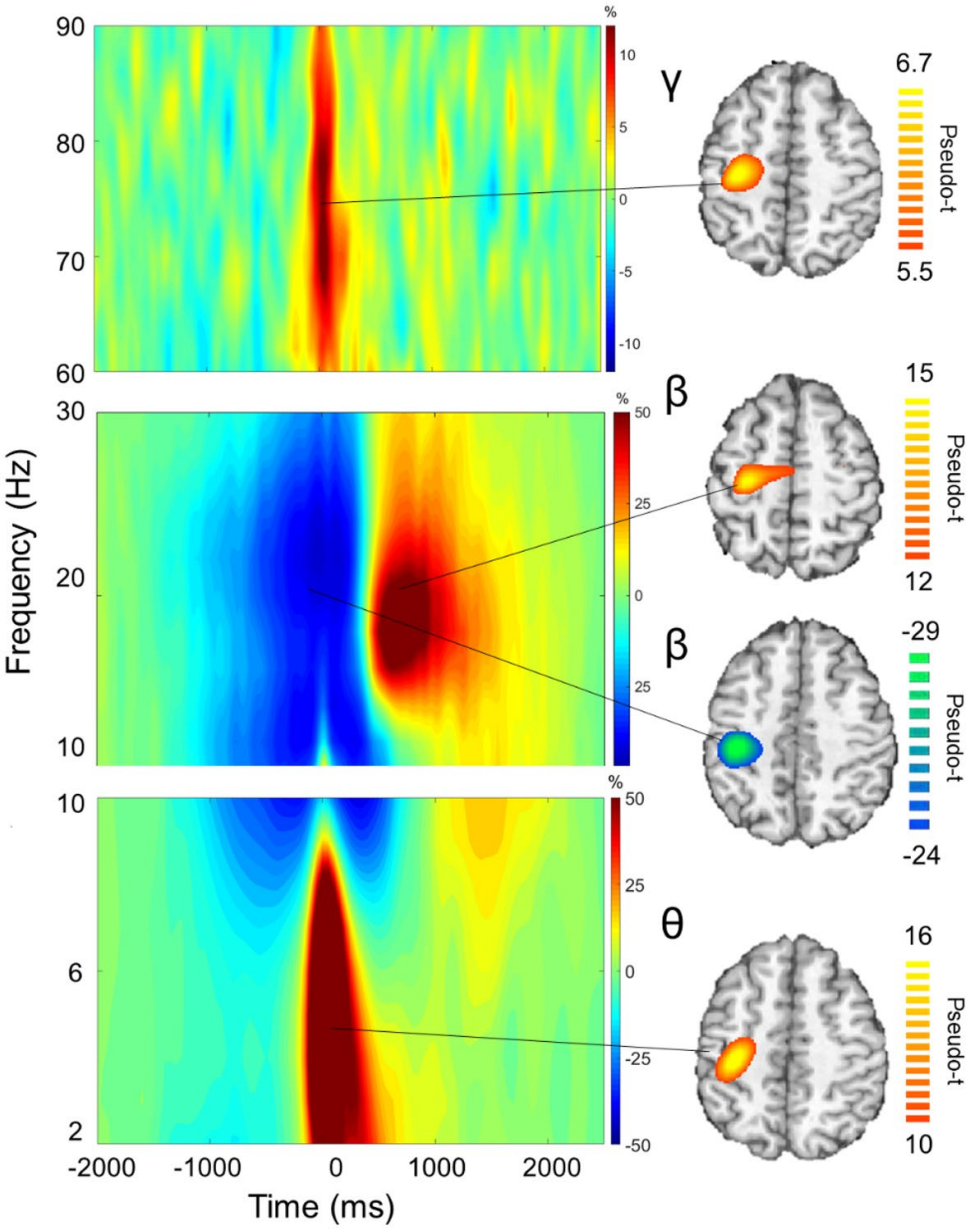
Sensor and source-level neural responses. Time frequency spectrograms for a sensor near the contralateral sensorimotor cortex (Left). The x-axis denotes time (ms) and y-axis denotes frequency (Hz). Onset of the button press occurred at 0 ms. The relative power of the spectrograms is expressed as a percent change from baseline (− 2000 to − 1500 ms time window). The significant oscillatory responses (*Ps* < 0.001, corrected) in the respective spectrograms were imaged and grand averaged across the respective task conditions and groups (Right). As shown in the beamformer images, the gamma event-related synchronization (ERS; 66–84 Hz), beta event-related desynchronization (ERD; 18–24 Hz), post-movement beta rebound (PMBR; 16–20 Hz) and theta ERS (4–6 Hz) were located in the motor “hand knob” region of the contralateral hemisphere. Group images for the participants with cerebral palsy and controls are shown in the supplementary material.

Visual inspection of the grand averaged oscillatory responses showed that there were notable changes in the gradiometers that spanned the contralateral fronto-parietal cortical region. Statistical analysis of the sensor level time–frequency spectrograms revealed significant theta ERS (4–6 Hz), beta ERD (18–24 Hz), PMBR (16–20 Hz) and gamma ERS (66–84 Hz) responses (*Ps* < 0.001, corrected; Fig. 3). Specifically, there was a prominent beta ERD that began 300 ms prior to the button press (i.e., 0 ms) and was sustained for approximately 200 ms (Fig. 3). Furthermore, there were gamma (0–100 ms) and theta (− 100 to 200 ms) ERS responses that coincided with the onset of the button press. There also was a PMBR in the 550–850 ms time window following the completion of the motor action.

**Figure 4:**
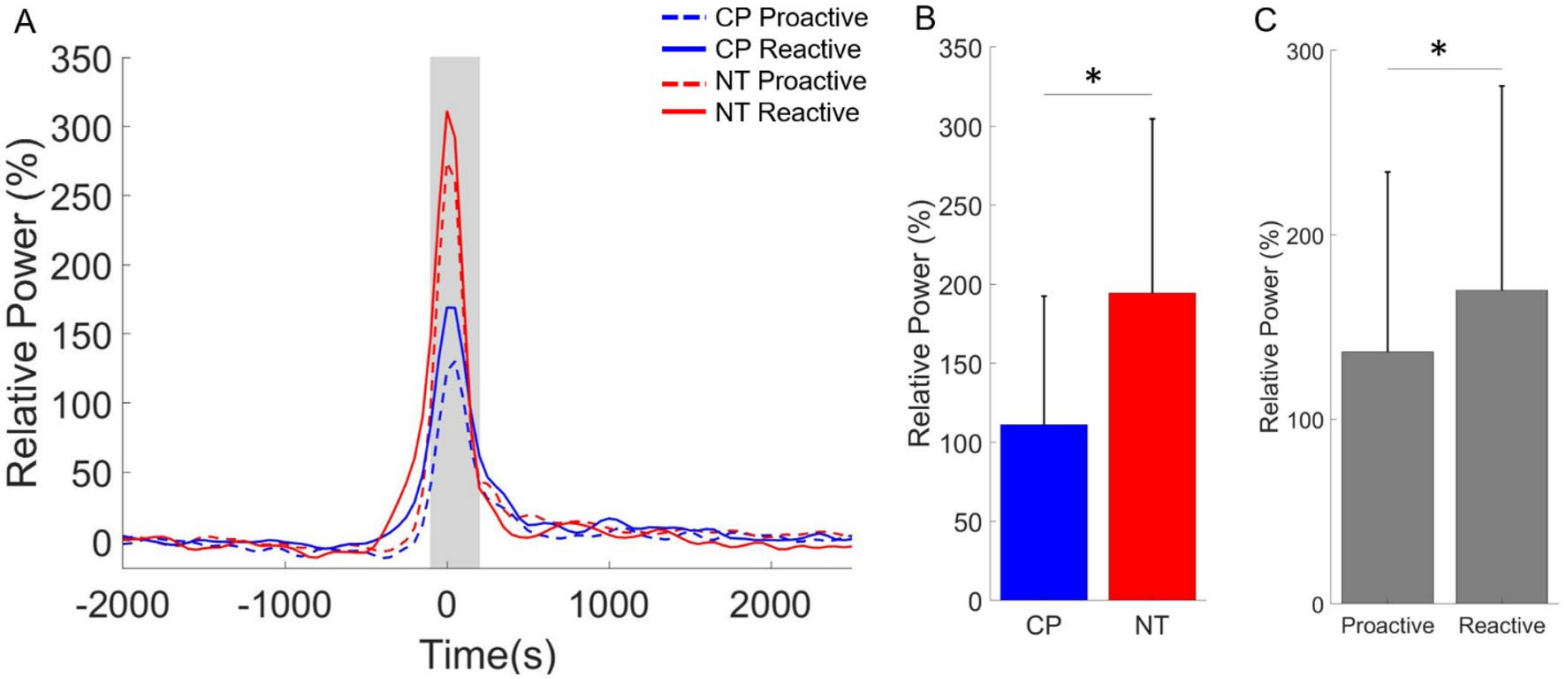
Theta event-related synchronization (ERS) in the sensorimotor cortices. (**A**) The neural time courses suggest that the theta (4–6 Hz) ERS was weaker for the adults with cerebral palsy (CP). The onset of the button press in the figure is at time 0 ms and the shaded area represents the time window where the theta ERS was imaged. (**B**) The main effect of group is shown in the bar graph. As shown, the theta ERS was notably weaker for the adults with CP when compared with the neurotypical (NT) controls. (**C**) The main effect of condition is shown in the bar graph. The theta ERS was stronger during the reactive condition across both groups. The bar graph results are shown as the mean ± the standard deviation. *p < 0.05.

We subsequently used a beamformer to image the respective cortical oscillations identified at the sensor level. All the oscillatory responses localized to the motor hand knob region of the contralateral hemisphere (Fig. 3). As detailed in the methods, we next extracted the neural time course from the peak voxel of the grand-averaged beamformer images and determined the average activity across the time windows of interest. Regarding the theta response, we detected a significant main effect of condition for the theta ERS (4–6 Hz) across the − 100 to 200 ms time window (Proactive = 136.58 (97.50) % (n = 37), Reactive = 169.89 (110.75) % (n = 37), F = 18.397, p = 0.0001; Fig. 4). Hence, indicating that the theta ERS was stronger for the reactive condition. There also was a main effect of group showing that the theta ERS was weaker overall for the adults with CP (CP = 111.26 (81.32) % (n = 36), NT = 194.54 (110.12) % (n = 38), F = 8.290, p = 0.007; Fig. 4B). The interaction of the group and condition was not significant (F = 3.75e − 5, p = 0.995).

**Figure 5:**
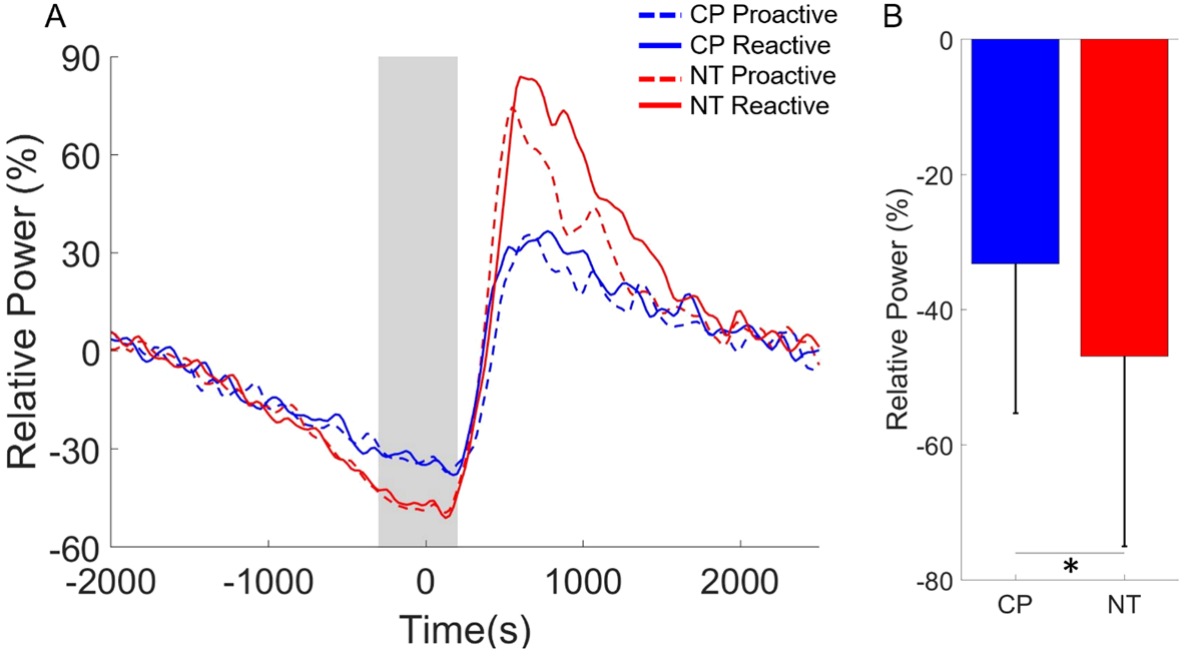
Beta event-related desynchronization (ERD) in the sensorimotor cortices. (**A**) The onset of the button press was at time 0 ms and the shaded gray area represents the time window where the beta ERD (18–24 Hz) was imaged. As shown, the beta ERD was weaker in the adults with cerebral palsy (CP) overall. Furthermore, the time courses appear to be similar across the proactive and reactive conditions. (**B**) Main effect of group is displayed in the bar graph where the beta ERD was significantly weaker in the adults with CP when compared with the neurotypical (NT) controls. The bar graph results are shown as the mean ± the standard deviation. *p < 0.05.

Our statistical analyses indicated there was a main effect of group for the beta ERD (18–24 Hz) during the − 300 to 200 ms time window (Fig. 5), which indicated that the beta ERD was weaker overall in the adults with CP (CP = − 33.19 (22.13) % (n = 38), NT = − 46.85 (28.17) % (n = 38), F = 5.846, p = 0.021; Fig. 5B). There was not a significant main effect of condition (Proactive = − 40.19 (18.31) % (n = 38), Reactive = − 39.85 (19.62) % (n = 38), F = 0.061, p = 0.807) or an interaction of condition and group (F = 0.160, p = 0.692).

**Figure 6:**
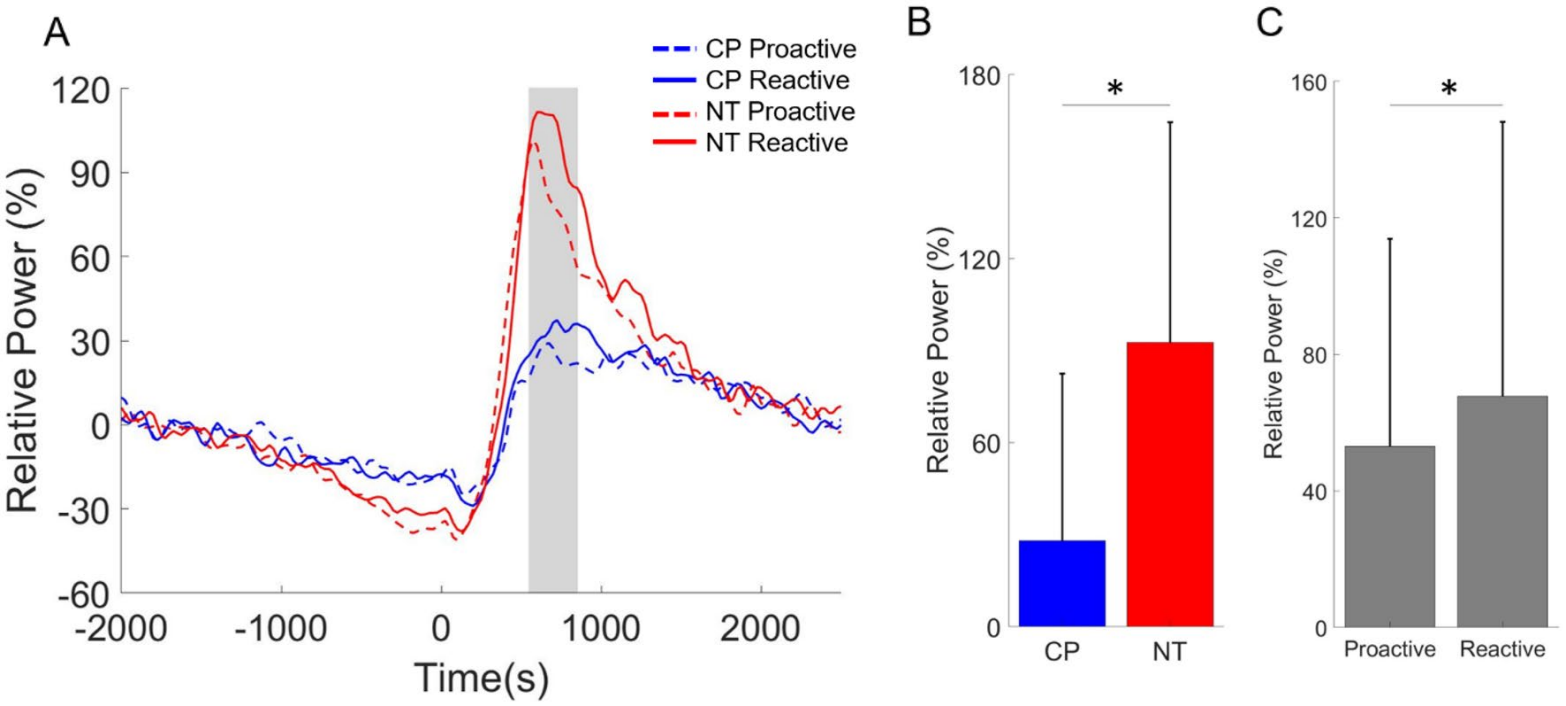
Post-movement beta rebound (PMBR) in the sensorimotor cortices. (**A**) Evaluation of the neural time course suggests that the PMBR was strongly diminished in the adults with cerebral palsy (CP). In addition, the PMBR was appreciably stronger during the reactive condition for both groups. The onset of the button press in the figure is at time 0 ms and the shaded gray area represents the time window where the PMBR was imaged. (**B**) Main effect of group is shown in the bar graph. As shown, the PMBR was weaker for the adults with CP when compared with the neurotypical (NT) controls. (**C**) The main effect of condition is shown in the bar graph. The PMBR was appreciably stronger during the reactive condition for both groups. The bar graph results are shown as the mean ± the standard deviation. *p < 0.05.

As per the PMBR response, there was a significant main effect of condition (Proactive = 52.99 (60.90) % (n = 35), Reactive = 67.68 (80.42) % (n = 35), F = 5.266, p = 0.028), indicating that the reactive condition had a stronger PMBR compared with the proactive condition (Fig. 6). There also was a main effect of group (CP = 28.05 (54.51) % (n = 36), NT = 92.63 (71.91) % (n = 34), F = 8.383, p = 0.007), showing that the PMBR was weaker overall for the adults with CP (Fig. 6B). The interaction term was not significant (F = 0.679, p = 0.416).

**Figure 7:**
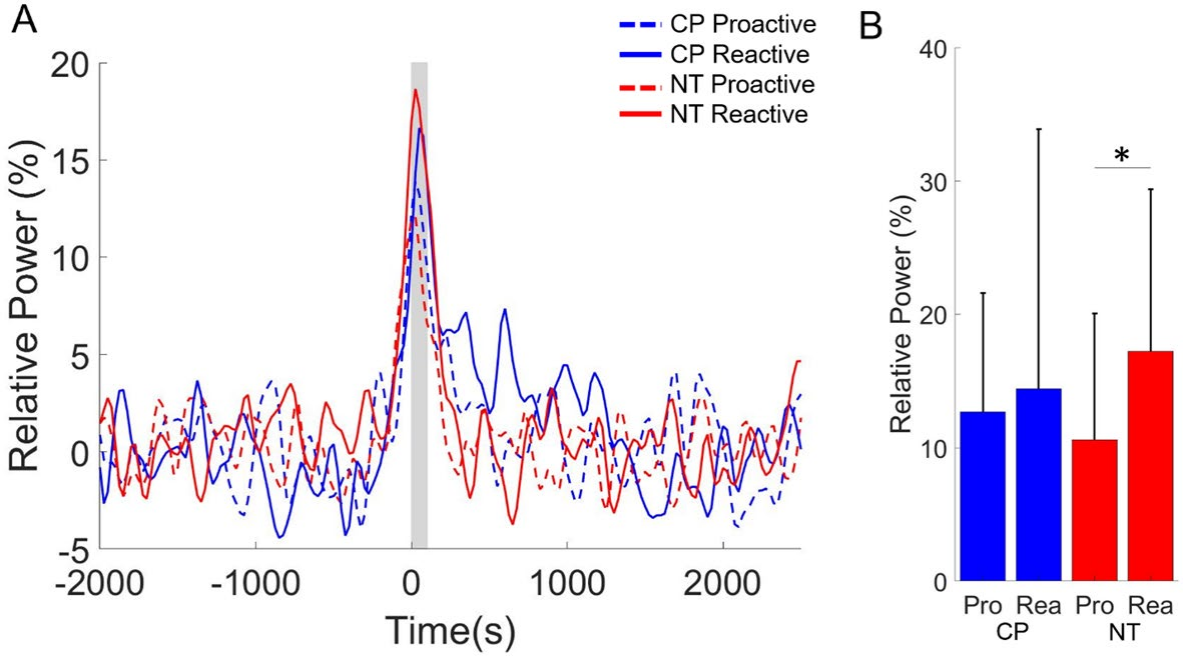
Gamma event-related synchronization (ERS) in the sensorimotor cortices. (**A**) The neural time courses for the gamma (66–84 Hz) event-related synchronization (ERS) did not appear different between the groups, but the gamma ERS was weaker for the proactive condition in neurotypical (NT) controls. The onset of the button press in the figure is at time 0 ms and the shaded gray area represents the time window where the gamma ERS was imaged. (**B**) As shown in the bar graphs, the gamma ERS was not significantly different between the adults with cerebral palsy (CP) and NT controls for the respective conditions. However, the gamma ERS was stronger for the NT controls during the reactive compared to the proactive condition, whereas this was not the case for the adults with CP. The bar graph results are shown as the mean ± the standard deviation. *p < 0.05 Pro = proactive, Rea = reactive.

For the gamma ERS (66–84 Hz) seen within the 0–100 ms time window, there was not a significant main effect of condition (Proactive = 11.67 (9.10) % (n = 36), Reactive = 15.81 (16.16) % (n = 36), F = 2.832, p = 0.102) or group (CP = 13.59 (15.08) % (n = 36), NT = 13.94 (11.24) % (n = 36), F = 0.182, p = 0.672). However, there was a significant interaction of condition and group (F = 4.257, p = 0.047; Fig. 7). Post-hoc analysis revealed that the reactive condition had a stronger gamma ERS for the NT controls when compared with the proactive condition (Proactive = 10.62 (9.45) % (n = 18), Reactive = 17.26 (12.14) % (n = 18), F = 6.471, p = 0.021, Fig. 7B). The reactive and proactive conditions were not significantly different in individuals with CP (Proactive = 12.71 (8.88) % (n = 18), Reactive = 14.44 (19.47) % (n = 18), F = 0.079, p = 0.782).

### Neuro-behavioral correlations

Regarding the neuropsychological assessments, participants with CP moved significantly less blocks for the Box and Blocks Test (CP = 43.19 (14.89) (n = 18), NT = 71.15 (18.55) (n = 19), p < 0.0001) and placed less pegs for the Purdue Pegboard Test (CP = 8.75 (4.77) (n = 18), NT = 15.37 (4.79) (n = 19), p < 0.0001) than NT. In addition, individuals with CP had significantly greater errors in the wrist position sense test (CP = 12.26 (3.61) (n = 18), NT = 8.01 (3.67) (n = 19), p < 0.0001).

**Figure 8:**
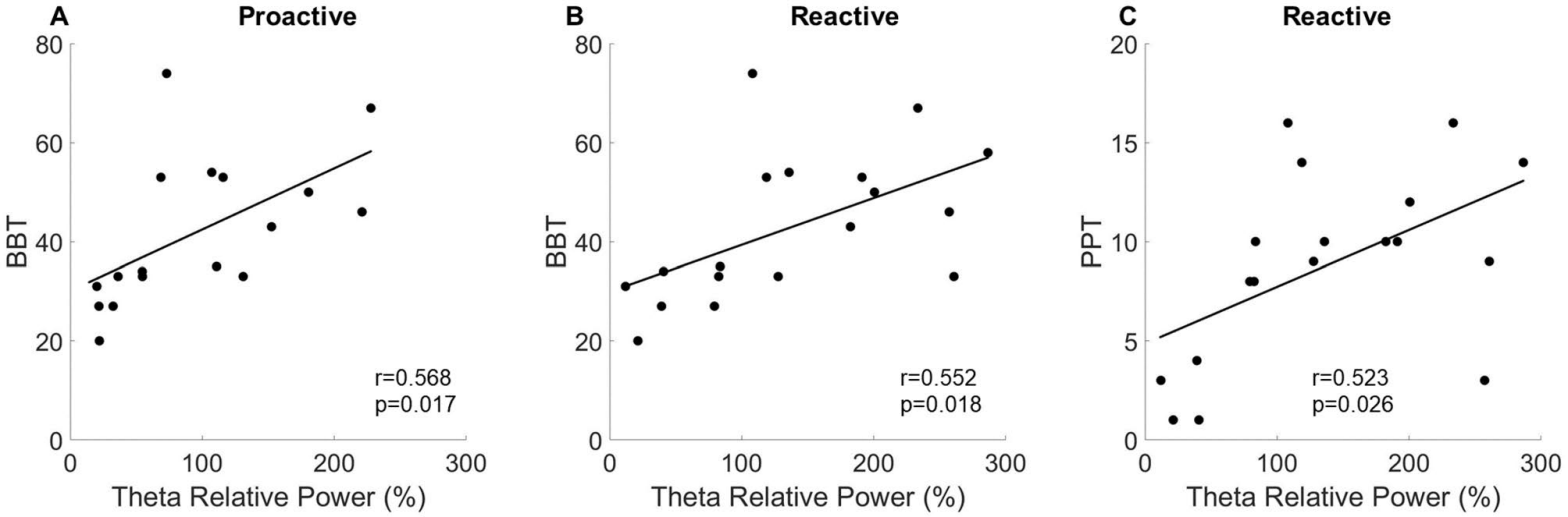
Relationships between strength of the theta event related synchronization (ERS) and the hand dexterity of the adults with cerebral palsy (CP). (**A**) The adults with CP who had a stronger theta ERS during the proactive (**A**) and reactive (**B**) conditions tended to move more blocks during the box and blocks test. Furthermore, the adults with CP who had a stronger theta ERS during the reactive condition also tended to place more pegs during the Purdue Peg Board Test (**C**).

For the adults with CP, there was a significant positive correlation between the strength of the theta ERS during the proactive (r = 0.568 (n = 17), p = 0.017, Fig. 8A) and reactive conditions (r = 0.552 (n = 18), p = 0.018, Fig. 8B) and scores on the Box and Blocks Test. There was also a significant positive correlation between the strength of the theta ERS in the reactive condition and the Purdue Pegboard scores in individuals with CP (r = 0.523 (n = 18), p = 0.026, Fig. 8C). These relationships imply that adults with CP who have a stronger theta ERS in either condition perform better on standardized tests of motor function. All other correlations for the adults with CP and NT controls were not significant (ps > 0.05).

## Discussion

The objective of this investigation was to use MEG brain imaging to evaluate potential differences in the sensorimotor cortical oscillations of adults with CP during an adaptive sensorimotor control task. Behaviorally, our results showed that the motor actions were slower in those with CP during the reactive trials. The behavioral differences seen in the adults with CP were accompanied by a weaker beta ERD, PMBR, and theta ERS. In addition, the strength of the gamma ERS was notably different during the reactive trials for the NT controls, but did not appreciably change between conditions for the adults with CP. Lastly, we found that the altered strength of the theta cortical oscillations seen in the adaptive sensorimotor task were partially connected to the altered hand dexterity seen in the adults with CP. Further discussion of the implications of these novel findings and their connection with the adaptive sensorimotor control of adults with CP are discussed in the following sections.

Overall, our results extend our understanding of the sensorimotor cortical oscillations that underlie the production of motor actions. For one, our results show that the strength of the gamma and theta ERS were stronger when the participant had to dynamically react to a change in the location and size of the target window. These results align with the outcomes from a prior study that used a similar experimental design^30^, which further implies that changes in the strength of the gamma and theta cortical oscillations are involved in reactively altering the initially planned motor action. Broadly, these sensorimotor gamma and theta oscillations are primarily assumed to reflect the execution of the motor command^15–17,63^. However, there is mounting evidence that the strength of the gamma ERS is influenced by interfering visual stimuli and selective attention^64–68^, suggesting that the strength of the gamma ERS has cognitive dependencies. Based on these studies, we contend that the stronger gamma ERS seen during the reactive condition reflects the heightened cognitive load or attention towards the changes that must be made to the motor command to meet the last second changes in the task demands. We infer that changes in the strength of the theta ERS for the reactive condition reflects changes in the timing of motor execution, as prior research has shown that the sensorimotor theta ERS is connected with the temporal structure of the motor execution^63^.

Independent of group, our results also identified that the PMBR was stronger during the reactive condition. Given that the PMBR occurs after the cessation of the movement, we suggest that the changes in the strength of the cortical oscillations are linked with the assessment of the movement fidelity following the performance. This premise is aligned with prior research showing that the strength of the PMBR is associated with assessment of the sensory feedback that is returned to the cortex after the motor action is complete^21,23,24^. As such, the altered PMBR seen during the reactive condition might reflect the amount of certainty in the adjustments that were made in the timing of the motor action when the expected motor task constraints were abruptly changed. Nevertheless, we should recognize that the PMBR results shown here are different from a prior study that found no difference between proactive and reaction task in terms of PMBR response^30^. However, this investigation only involved NT controls; therefore, the addition of individuals with CP may be the reason for the differences noted here.

The adults with CP also had a weaker PMBR compared to NT controls for both conditions. This result is aligned with prior studies that have also identified that the PMBR is weaker in persons with CP ^24,25^. As highlighted previously, the PMBR has been associated with updating of the internal model after the sensory feedback is returned. Altogether, these combined results suggest that the altered PMBR seen for the persons with CP might indicate greater uncertainty about the sensory feedback after the completion of the motor task^23,24^. We speculate that this uncertainty is likely related to the altered sensory processing noted across the behavioral^1,69–72^ and neurophysiology literature for persons with CP^13,62,73–78^. As such, the altered sensory processing at both the cortical and spinal cord level likely has cascading effects on the sensorimotor system and affects the fidelity assessment of adjusted motor outcomes.

The sensorimotor beta ERD for the adults with CP was weaker when compared to the NT controls. These results are well aligned with prior research, which has shown that youth with CP have deviant sensorimotor beta cortical oscillations, and that these altered cortical oscillations are connected with their uncharacteristic motor actions and motor production errors^25^. Given that there was a lack of condition-wise differences seen for both the adults with CP and NT controls, it is possible that the noted difference in the strength of the beta ERD is not impacted by the need to abruptly change in the timing of the motor command. Rather, the strength of the beta ERD reflects the overall certainty of the motor plan, as there should be certainty in the basic tenants since the task design involved visually attending to a dot as it approached the target zone where the known motor action should be executed. As such, the participants knew far in advance when they would likely press the button. We contend that the altered strength of the beta ERD seen in the adults with CP more likely reflects the uncertainty in sufficiently exciting the sensorimotor cortical neurons to rapidly press the button rather than the certainty of the task dynamics to be completed, per se. Given that the time window of interest spanned the motor planning and execution states, it is alternatively plausible that the weaker beta ERD might just reflect the reduced number of neurons that can be excited due to the developmental brain injury. This notion is partly aligned with a prior transcranial magnetic simulation (TMS) study that revealed persons with CP lack the ability to modulate the excitability of the sensorimotor cortical neurons ^79^.

Our results identified that the strength of the gamma ERS was not different between the proactive and reactive conditions for the adults with CP, but there was a condition-wise difference for the NT controls. As stated previously, several prior studies have found that the strength of the gamma ERS is influenced by higher order cognitive processes^64,66–68,80^. Given that the general tenets of the motor actions to be completed for both conditions were the same, the differences in gamma ERS seen for the controls implies that the gamma ERS reflects the heightened cognitive demand or attention involved in altering the timing of the motor action on-the-fly to match the updated task demands. The lack of a change in the gamma ERS seen in the adults with CP suggests that they were less capable of cognitively adapting the timing of their motor action during the reactive condition. Alternatively, it could be that generating rapid motor actions might be challenging for adults with CP regardless of the task condition. We suspect that these challenges might be related to spasticity and/or the inability to activate the Type II fast-twitch alpha motor neurons^81^. Taken together, these data suggest that adults with CP might not be able to adapt as well to last-second changes in the motor task demands.

Across both conditions, the theta ERS for the adults with CP was also weaker when compared to the NT controls. Furthermore, adults with CP who had a weaker theta ERS also tended to have poorer performance on the standardized motor assessments. Together, these results suggest that alterations in the motor-related theta oscillations are linked with the hand dexterity of adults with CP. We infer that this connection reflects an inability to properly regulate the temporal structure of motor execution^63^. It is alternatively possible that the weaker theta ERS reflects deficiencies in exciting these pathways, as a prior TMS study has shown that persons with CP lack the ability to modulate the excitability of the corticospinal pathways^79^. On the other hand, it is possible that those who could perform the MEG task better had more theta power because the theta band response is phase-locked to the onset of the button press. Although these explanations are plausible, fewer studies have evaluated motor-related theta ERS responses in those with CP. As such, further investigations are necessary to illuminate the connection between the aberrations in theta oscillations and the uncharacteristic motor actions seen in adults with CP.

## Limitations

As with any investigation of persons with CP, there was heterogeneity in our patient sample. Thus, it is possible that there are individual differences due to the perinatal brain lesions. Based on our small sample size, we are unable to evaluate how differences in the perinatal brain injuries may have impacted the strength of the respective sensorimotor cortical oscillations. That being said, care should be taken when attempting to connect such structural alterations to functional deficits as there are many studies showing that structural brain aberrations do not frequently predict the sensorimotor deficiencies^82–88^. The reason is that there is an enormous potential for experience and environmental factors to instigate beneficial and/or detrimental neuroplastic changes that impact the long-term sensorimotor presentation. As such, some individuals with CP can have no notable brain insults on their MRI, yet have significant sensorimotor presentations^82–88^. The opposite is also true in that individuals with CP can have substantial brain insults, but the insult appears to have less of an impact on their sensorimotor presentations. There still is a notable knowledge gap in our understanding of the structure–function connection in persons with CP. An additional limitation is that our understanding of the sensorimotor control of the hand motor actions was based on a simplified button-press task. Performing a button-press is obviously very different from the dexterous motor actions that can be performed by the hand. That being said, the button press task was used as a surrogate for reveling how the classic sensorimotor cortical oscillations are perturbed in adults with CP when the task demands are changed on the fly. It should be highlighted that our conjectures between alterations in the sensorimotor cortical oscillations and the hand dexterity clinical assessments were associative and not causal.

## Conclusion

This study found that individuals with CP exhibit atypical sensorimotor cortical oscillations during an adaptive sensorimotor control task. Specifically, the strength of the gamma ERS was different during the reactive trials for NT controls but did not significantly change for adults with CP between conditions. This may imply that adults with CP were less capable of cognitively adapting the timing of their motor action during the reactive condition. Secondarily, the adults with CP had a weaker beta ERD, PMBR and theta ERS. Lastly, better hand dexterity on the clinical assessments appeared to be linked with a stronger theta ERS in the adults with CP. Based on the results shown here, we speculate that employing unexpected changes in the motor goal might enhance the upper extremity adaptability of persons with CP during physical or occupational therapy. An exemplary approach would be to slightly change the location of the target prior to the initiation of the reach. Hence, requiring the participant to update their feedforward motor command. This approach could be implemented with a touchscreen technology (i.e., Bioness Integrated Therapy System) or reaction training lights (i.e., BlazePods, ROX). We suspect that this neuroscience informed treatment approach might result in greater clinical gains that transfer to the real-world and are not restricted to performance in the clinical environment.

### Supplementary Information

Supplementary Information.

## Data Availability

The data that support the findings of the study are available from the corresponding author upon reasonable request.
