# Supplementary material for: Adults with cerebral palsy exhibit uncharacteristic cortical oscillations during an adaptive sensorimotor control task
Source: Sci Rep. 2024 May 11;14:10788. doi: 10.1038/s41598-024-61375-x (PMC11088662; doi:10.1038/s41598-024-61375-x)

| Subject Number | Age (yrs.) | Sex | Presentation | GMFCS | MACS | MRI Radiologic Read |
| --- | --- | --- | --- | --- | --- | --- |
| M68102369 | 24 | M | Diplegia | II | II | Partial absence of the corpus callous, aqueductal stenosis |
| M68108902 | 60 | M | Diplegia | I | III | No abnormal findings |
| M68113991 | 38 | M | Hemiplegia | IV | IV | Bilateral posterior parietal periventricular volume loss and diffuse abnormal white matter signal, remote infarcts and atrophy of the posterior corpus callosum |
| M68115584 | 21 | M | Hemiplegia | I | I | Mild heterogenous decreased signal involving the bilateral periatrial trigones, and sequela of periventricular leukomalacia |
| M68119585 | 23 | M | Hemiplegia | I | I | Periatrial trigone consistent with remote ischemia |
| M68126344 | 26 | M | Hemiplegia | II | III | Right cerebral hemisphere polymicrogyria, and mild asymmetric ex vacuo enlargement of the right lateral ventricle |
| M68126930 | 19 | F | Diplegia | III | I | White matter signal abnormality and volume loss |
| M68127498 | 29 | F | Hemiplegia | I | I | Areas of abnormal white matter signal associated with a sequela of prior ischemia |
| M68135290 | 40 | F | Diplegia | IV | III | Incomplete hypoplasia of the posterior body of the corpus callosum |
| M68136698 | 33 | F | Diplegia | I | I | No abnormal findings |
| M68153097 | 32 | M | Diplegia | I | II | Prominence of the occipital horns of the lateral ventricles with underlying parietal and occipital atrophy and marked thinning of the corpus callosum |
| M68156820 | 22 | M | Diplegia | III | III | Severe corpus callosal thinning. severe posterior white matter atrophy and parietal cortical atrophy |
| M68157181 | 47 | F | Diplegia | III | III | No abnormal findings |
| M68165366 | 31 | F | Hemiplegia | I | I | Unable to obtain MRI |
| M68172923 | 28 | M | Diplegia | II | I | Unable to obtain MRI |
| M68174936 | 39 | F | Diplegia | III | III | White matter signal abnormality and volume loss |
| M68192112 | 50 | F | Diplegia | I | I | Right paracentral disc extrusion at C5-6 with severe spinal canal stenosis |

**Table S1.** Demographics and MRI radiologic reads for the participants with cerebral palsy. M= Male, F= Female, GMFCS= Gross Motor Function Classification Score, MACS = Manual Ability Classification Score.

| M68199258 | 23 | F | Diplegia | II | II | Cerebellar hypoplasia/dysplasia with marked asymmetric enlargement of the 4th ventricle. Bilateral supratentorial polymicrogyria most prominent in the occipital and parietal lobes. Callosal dysgenesis with absence of the splenium. |
| --- | --- | --- | --- | --- | --- | --- |
| M68199688 | 35 | M | Diplegia | II | II | No abnormal findings |
|  |  |  |  |  |  |  |

**Figure S1.** Group beamformer images for the theta event related synchronization (ERS; 4-6 Hz), beta event-related desynchronization (ERD; 18-24 Hz), post-movement beta rebound (PMBR; 16-20 Hz). These oscillatory responses, unlike gamma, were found to be significantly different between the respective groups. As shown, the imaged oscillatory activities were located in the same cortical region of the contralateral hemisphere in each group.


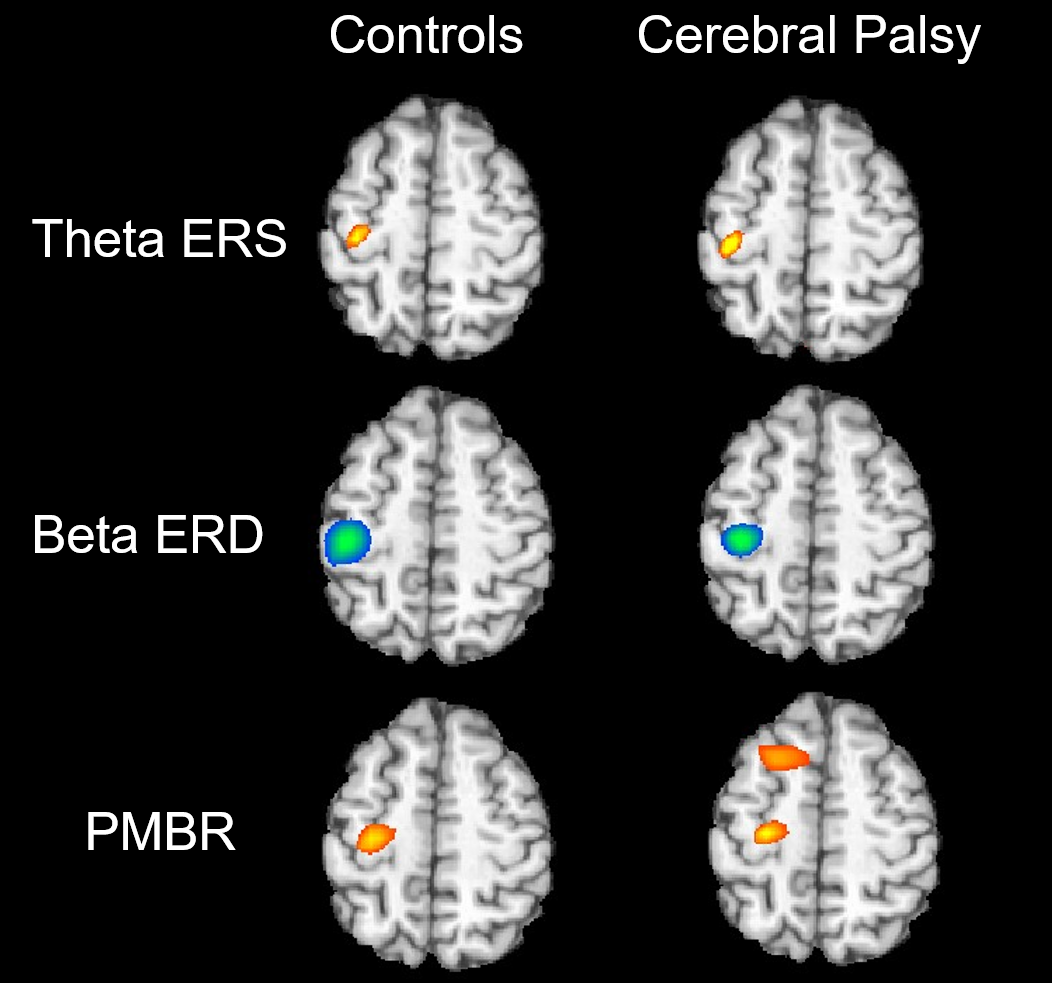

Supplement: Supplementary file 1 — Supplementary Information. [file 41598_2024_61375_MOESM1_ESM.docx]
